# Raising the Oxidation Resistance of Low-Alloyed Mg-Ca Alloys Through a Preheating Treatment in an Argon Atmosphere

**DOI:** 10.3390/ma17225481

**Published:** 2024-11-10

**Authors:** Siyuan Liu, Jonathan Apell, Zhihui Liu, Guojun Liu, Xingyou Lang, Yongfu Zhu, Qing Jiang

**Affiliations:** 1 Key Laboratory of Automobile Materials, Ministry of Education (Jilin University), School of Materials Science and Engineering, Jilin University, 5988# Renmin Street, Changchun 130022, China; 15754314975@163.com (S.L.); liuguojun@jlu.edu.cn (G.L.); xylang@jlu.edu.cn (X.L.); jiangq@jlu.edu.cn (Q.J.); 2 Institute of Materials Science and Engineering, Chemnitz University of Technology, Erfenschlager Straße 73, 09125 Chemnitz, Germany

**Keywords:** magnesium, oxidation resistance, oxide layer, heat treatment

## Abstract

With the rise and development of aerospace, communications, electronics, medical, transportation and other fields, magnesium (Mg) and its alloys have attracted much attention for their high specific strength and stiffness, good electromagnetic shielding properties, excellent damping properties and other advantages. However, magnesium has a high affinity for oxygen, producing magnesium oxide (MgO), and MgO’s Pilling–Bedworth ratio (PBR) of 0.81 is not protective. The occurrence of catastrophic oxidation is unavoidable with the increase of oxidation time and temperature. A promising approach is to perform an appropriate pretreatment in conjunction with alloying to obtain a dense and compact composite protective film. In this work, the effect of a preheating treatment on the oxidation resistance (OR) of Mg-*x*Ca (*x* = 1, 3 and 5 wt. %) was investigated. The preheating was carried out in an Ar atmosphere at 400 °C for 8 h. Upon it, a dense and compact MgO/CaO composite protective film was formed on the surface, which is CaO-rich especially in the vicinity to the surface. The alloys’ oxidation resistance was strongly increased due to the composite protective film formed during the preheating treatment, in particular for Mg-3Ca. Relative to the Mg-hcp phase, the OR of the Mg_2_Ca phase was significantly raised.

## 1. Introduction

In recent years, magnesium (Mg) alloys have been gradually becoming important light-weight structural materials for aerospace and rail transportation due to their low specific density, high specific strength and good vibration reducing performance [1,2,3]. It has been shown that the use of magnesium alloys instead of steel can effectively reduce weight in automotive applications. Under the new situation of increasingly strict automobile emission regulations and more urgent energy saving and consumption reduction, the world’s automobile industry has turned its attention to magnesium alloys. It is believed that Mg is a better light material than aluminum alloy and plastic since it has a more pronounced weight reduction effect, more effective energy saving and consumption reduction, and more favorable environmental protection [4]. Magnesium and its alloys are the lightest industrial metals, but they have a high affinity for oxygen and easily oxidize in air. The Pilling–Bedworth ratio (PBR) value of magnesium oxide (MgO) is 0.81, which refers to a loose and porous structure [5,6,7]. As the oxidation reaction progresses (especially at high temperatures), cracks may form in the oxide layer. This reduces oxidation resistance (OR) and severely limits the use of Mg and its alloys in different applications [8].

Based on previous studies on the oxidation of Mg alloys [9,10,11,12], alkaline metals and rare earth metals have been reported as alloying elements for improving the OR of Mg alloys. Wu et al. [13] pointed out that the addition of Er significantly improves the OR of Mg, attributed to the formation of loose MgO and Er_2_O_3_ in the intermediate layer and the formation of dense fine-grained Er_2_O_3_ in the inner layer. Lin et al. [14] found that the addition of 0.25 wt.% Ce to both AZ91 and AM50 alloys increased their ignition temperatures by 50 °C due to the formation of Ce_2_O_3_ with a PBR of 1.16 on the surface. When sufficient amounts of Al are added for alloying, the β-phase (Mg_17_Al_12_) is formed as an isolated phase, as eutectic, or as precipitates [15]. However, due to the poor thermal stability (the melting temperature of the β phase is 437 °C), the β phase is prone to oxidation with increasing temperature, which limits the practical application at high temperatures [16]. Zinc (Zn) is known to improve the corrosion resistance of Mg alloys by reducing the levels of impurities in them [11]. However, Mg-Zn inter-metallics have significant evaporation pressure and show selective oxidation at high temperatures [17]. Considering that the mechanical properties are usually weakened by increasing solute concentration, the OR of magnesium alloys should be improved by reducing the content of alloying elements [18].

When it comes to alloying elements for improving the OR, Ca is one of the most effective candidates because the addition of Ca can effectively improve the OR and the ignition point of Mg alloys [1,4,19,20,21]. You et al. [5] reported that a porous and loose MgO layer formed on the surface of pure magnesium after oxidation in air at 440–500 °C for up to 7 h, whereas a mixed oxide layer of MgO and CaO was observed in the Mg-3Ca alloys. Cheng et al. [20] reported that the addition of 1.5 wt.% Ca to AZ91 alloy ensured that no significant oxidation was observed after 7 h of exposure at 400 °C. Li et al. [22] increased the ignition temperature of AZ91 to 325 °C by adding 6 wt.% Ca. Fan et al. [23] proposed that Ca has a third element effect in Mg-3.5Y-0.8Ca alloys to enhance the surface activity of Y in the alloys, which significantly reduces the critical content of Y required for the formation of an intact Y_2_O_3_ protective film in Mg-Y alloys from 10 wt.% to 3.5 wt.%. Besides those alloying elements, the preheating treatment method may also have an effect on the OR of Mg alloys [24,25], where alloying elements with a high affinity for oxygen may form a protective film on the surface of the alloy [26,27]. Our previous study showed that the OR of Mg-CaO alloys can be significantly improved by the preheating treatment [28]. Mg_2_Ca was not observed for the investigated composition range, which could be attributed to the residual small amount of O in the system of Mg-CaO-O we studied [28]. Meanwhile, previous studies have shown that Mg_24_Y_5_, Al_2_Y and Mg_5_Gd are typical intermetallic compounds in the Mg-2Y-*x*Be, Mg-2.5Y-*x*Al and Mg-3.5Gd-*x*Ca systems, and the formation of these kinds of compounds from the dissolved solutes, such as Y and Gd, has a significant influence on the OR [29,30,31]. Therefore, it is extremely valuable to investigate the effect of intermetallic compounds on the OR of the preheated Mg-*x*Ca alloys.

In the present study, the effect of the preheating treatment at 400 °C for 8 h in a high purity Ar atmosphere (99.9999%) on the OR of Mg-*x*Ca (*x* = 1, 3 and 5 wt.%) is investigated. Attention is given to the formation of the thin surface protective layer and its effect on the OR. The role of the eutectic phase in the OR is also discussed.

## 2. Experiment

### 2.1. Experimental Materials and Methods

**Figure 1:**
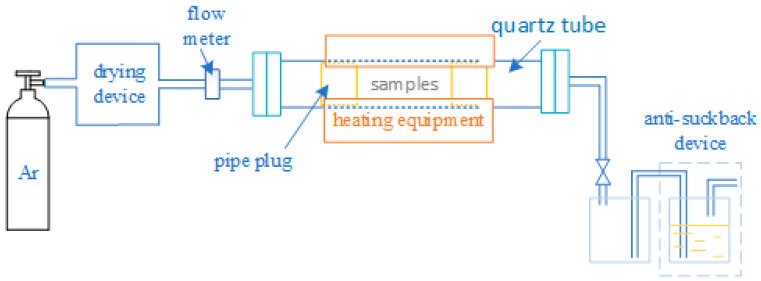
Preheating treatment experimental installation diagram.

Mg-*x*Ca alloys (*x* = 1, 3 and 5 wt.%) were melted and cast in an electric resistance furnace under shielding gases (SF_6_ and CO_2_) using a graphite crucible and mold, respectively. The alloy ingots were cut and hot rolled into 0.5 mm thick sheets, followed by mechanical and electropolishing, where the electropolishing was carried out in a 1:2 mixture of phosphoric acid and ethanol. The specimens for the oxidation tests were created by punching into discs with a diameter of 5 mm. Subsequently, Mg-*x*Ca alloys were ultrasonically cleaned in acetone and distilled water, respectively. In order to carry out comparative experiments, pure Mg samples were prepared by applying the above experimental steps. Before conducting the oxidation experiment, Mg-*x*Ca alloys were preheated in a high-purity Ar atmosphere (99.9999%) at approximately 0.1 Pa oxygen partial pressure with the experimental installation shown in Figure 1. Prior to the preheating treatment, the samples were placed in the middle of the quartz tubes in the position of the heating unit, and the quartz tubes were air-washed (four times) with a vacuum pump, where the atmospheric pressure inside the quartz tubes was pumped down to about 0.1 MPa during each air washing process. The preheating treatment was carried out at 400 °C for 8 h to ensure that Ca segregated to the surface and Mg did not undergo catastrophic oxidation.

### 2.2. Material Characterization

After the preheating treatment, the oxidation tests were performed at a flow rate of 60 mL/min for 2 h at 400 °C in an atmosphere of pure oxygen (0.1 MPa). The samples were heated at a rate of 50 °C/min until the experimental temperature reached 400 °C. The mass gain of pure Mg, not preheated Mg-*x*Ca (uP-Mg-*x*Ca) and preheated Mg-*x*Ca (P-Mg-*x*Ca) was measured by thermogravimetry (METTLER 1100LF, Mettler Toledo, Zurich, Switzerland ) with a high sensitivity of 0.001 mg. Oxidation morphologies of pure Mg, uP-Mg-*x*Ca and P-Mg-*x*Ca alloys were analyzed by thermal field emission scanning electron microscopy (FESEM, JEOL, Tokyo, Japan). The surface morphology and cross-section thickness of the oxide layer were analyzed by scanning electron microscopy (SEM, TESCAN, Brno, Czech Republic) and transmission electron microscopy (TEM, JEOL, Tokyo, Japan). For the protective layer thickness (Section 3.1), multiple measurements (10 times) were taken along the TEM lamella for the Mg region and the Mg_2_Ca region. TEM cross-sectional specimens were prepared using FIB-SEM double-beam electron microscope technique equipped with a Ga liquid metal ion source (FEI helios G4 CX, Thermo Scientific, Waltham, MA USA) with accelerating voltages up to 30 kV and currents from 0.6 pA to 65 nA by means of lifting and grooving techniques. First, two thin platinum coatings were deposited over the desired area as a protective film. Before finishing the production of the TEM foil test samples, foils with a thickness of ~100 nm were carefully polished several times at low accelerating voltages of 5 kV and 48 pA. Phase analysis was carried out by 40 kV and 300 mA Cu Kα radiation X-ray diffraction (XRD, Rigaku, Tokyo, Japan). Elemental composition on the surface was determined by XPS (ESCALAB 250Xi, Thermo Scientific, Waltham, MA USA) technique with Ar ion gun.

## 3. Results and Discussion

### 3.1. Microstructure of uP-Mg-xY and P-Mg-xY Alloys

**Figure 2:**
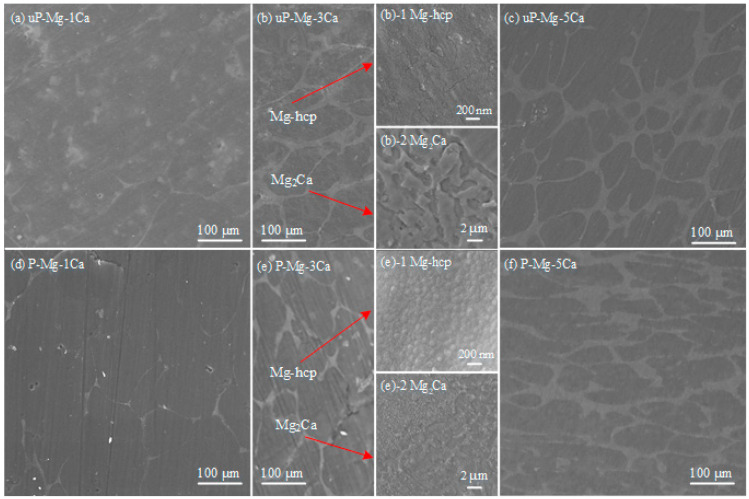
Surface morphologies of uP-Mg-*x*Ca and P-Mg-*x*Ca alloys preheating at 400 °C for 8 h in Ar with uP-Mg-1Ca in (**a**), uP-Mg-3Ca in (**b**), uP-Mg-5Ca in (**c**), P-Mg-1Ca in (**d**), P-Mg-3Ca in (**e**) and P-Mg-5Ca in (**f**). The inserts in (**b**) uP-Mg-3Ca and (**e**) P-Mg-3Ca, respectively, are high magnification FESEM images of the Mg-hcp phase in (**b**)-1 and (**e**)-1 and the eutectic phase Mg_2_Ca in (**b**)-2 and (**e**)-2.

Figure 2 shows the SEM surface morphologies of the uP-Mg-*x*Ca and P-Mg-*x*Ca alloys after the preheating treatment at 400 °C for 8 h in (a–c) and (d–f), respectively. In the not preheated Mg-*x*Ca alloys, Mg-hcp phase of Mg-Ca can be observed with a dark contrast in a granular shape. In the intergranular spacing, the eutectic phase Mg_2_Ca is found [11,21]. Mg_2_Ca phase forms in a eutectic reaction (liquid into Mg-hcp + Mg_2_Ca) that occurs due to the increasing Ca composition in the melt during cooling.

After the preheating treatment (Figure 2d,e), the surface of P-Mg-*x*Ca is flat and dense; in Figure 2d, tiny pores are observed for P-Mg-1Ca. Consistent with the phase diagram, the eutectic phase Mg_2_Ca is still found in the P-Mg-*x*Ca alloys. The inserts in (b) uP-Mg-3Ca and (e) P-Mg-3Ca, respectively, show high magnification FESEM images of the Mg-hcp phase in (b)-1 and (e)-1 and the eutectic phase Mg_2_Ca in (b)-2 and (e)-2. The surface of Mg-hcp phase in uP-Mg-3Ca in the insert of Figure 2(b)-1 seems flat but with some tiny cracks formed on it. In contrast, the surface of the P-Mg-3Ca is much denser and covered by tightly connected small grains, as shown in Figure 2(e)-1. Thus, it is expected that the surface of the preheated Mg-*x*Ca should provide more protection than that of the not preheated ones, based on a literature work on the preheated Mg-*x*CaO alloys [28]. Note that the presence of tiny pores in P-Mg-1Ca in Figure 2d may be harmful to its OR, and its formation may be related to the low amount of Ca that is not enough to compensate the negative impact of the low PBR of MgO even after the preheating treatment. In addition, the Mg_2_Ca eutectic phase in uP-Mg-3Ca in Figure 2(b)-2 consists of a discontinuous protruding structure, while such a structure mostly vanishes after preheating, as shown in Figure 2(e)-2.

**Figure 3:**
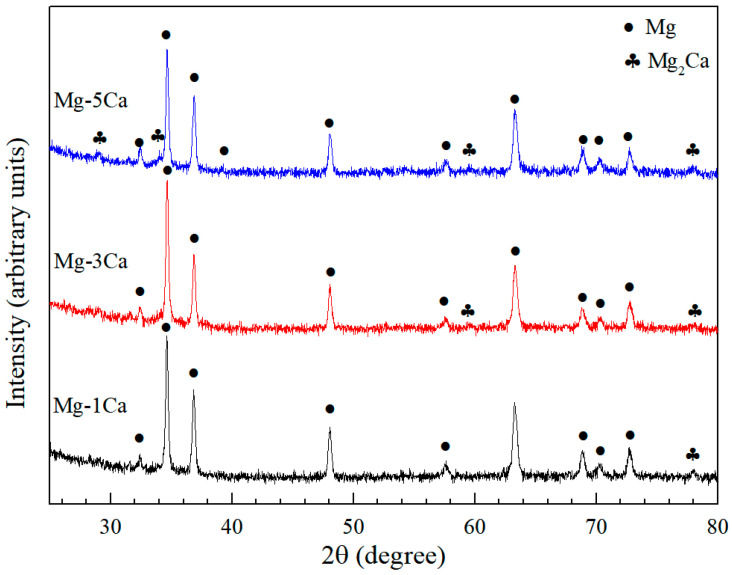
XRD results of the uP-Mg-*x*Ca alloys. It shows the formation of Mg_2_Ca as eutectic phase together with Mg-hcp.

To analyze the phases present in Mg-*x*Ca in Figure 2, Figure 3 shows the XRD patterns of the uP-Mg-*x*Ca alloys, where α-Mg as the major phase and the eutectic phase Mg_2_Ca are clearly detected in those three alloys. Meanwhile, the number of Mg_2_Ca reflections rises slightly with the increase in the Ca content. This further demonstrates that the eutectic phase distributed in a network in Figure 2 is composed of Mg_2_Ca.

**Figure 4:**
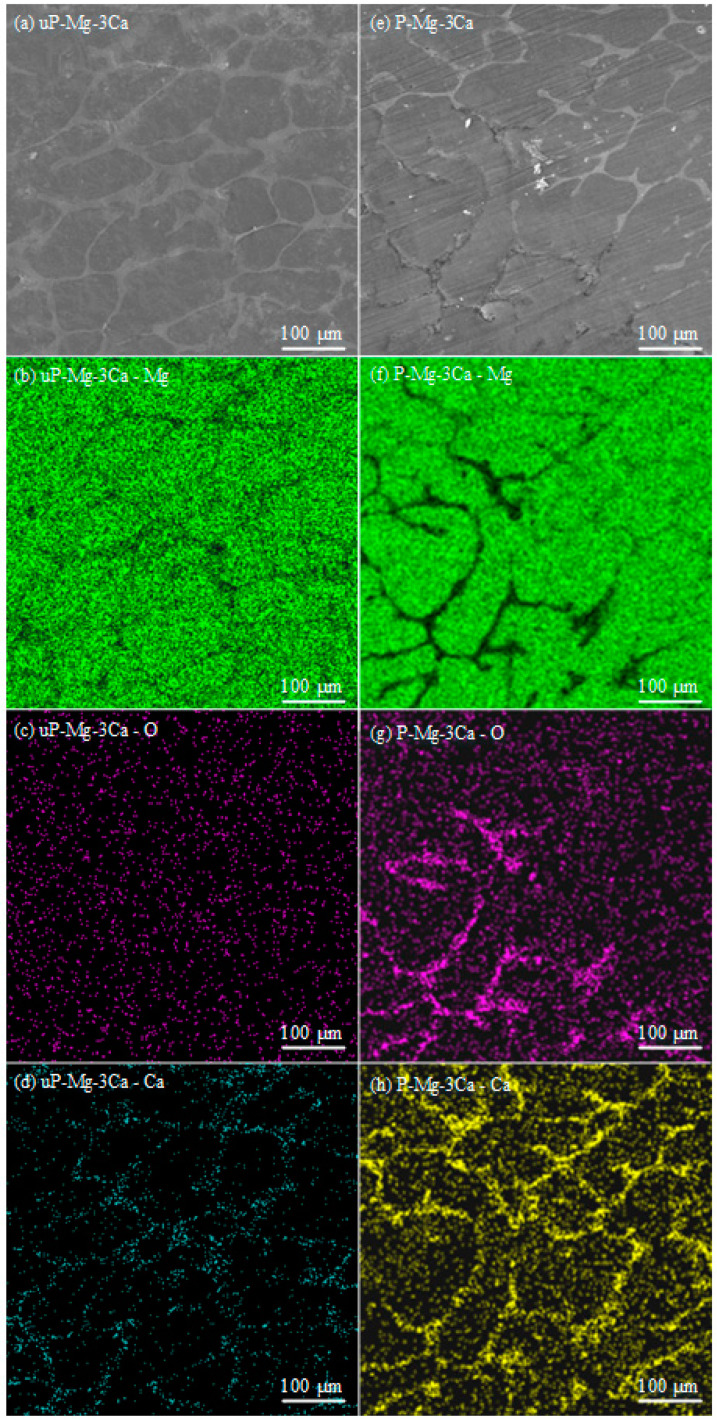
EDS mapping results of uP-Mg-3Ca and P-Mg-3Ca preheating at 400 °C for 8 h. (**a**) uP-Mg-3Ca surface with element distribution of (**b**) Mg, (**c**) O and (**d**) Ca; (**e**) P-Mg-3Ca at 400 °C surface with element distribution of (**f**) Mg, (**g**) O and (**h**) Ca. It shows the preferential oxidation of the Ca-rich Mg_2_Ca phase.

Figure 4 shows the surface morphology SEM images of uP-Mg-3Ca and the corresponding EDS mappings of Mg, Ca and O in (a–d) and P-Mg-3Ca in (e–h). Similar to uP-Mg-3Ca in Figure 4a, the eutectic Mg_2_Ca phase can also be observed for P-Mg-3Ca in Figure 4e. The EDS mapping results show that the intensity of the Mg signal of uP-Mg-3Ca and P-Mg-3Ca in Figure 4b,f is the highest. For uP-Mg-3Ca, the signals of O in Figure 4c and Ca in Figure 4d can be observed but are somewhat weak. In the regions where the eutectic phase Mg_2_Ca is located, the signals of Ca and O are relatively stronger. Relative to uP-Mg-3Ca, the signals of O in Figure 4g and Ca in Figure 4h show a significant increase after the preheating, especially in the regions where the eutectic phase Mg_2_Ca is located. This shows the preferential oxidation of the Ca-rich Mg_2_Ca phase, and the increase in the intensities of Ca and O in the α-Mg phase is attributed to outward segregation of Ca during the preheating.

### 3.2. TEM Analysis of P-Mg-3Ca Alloy

**Figure 5:**
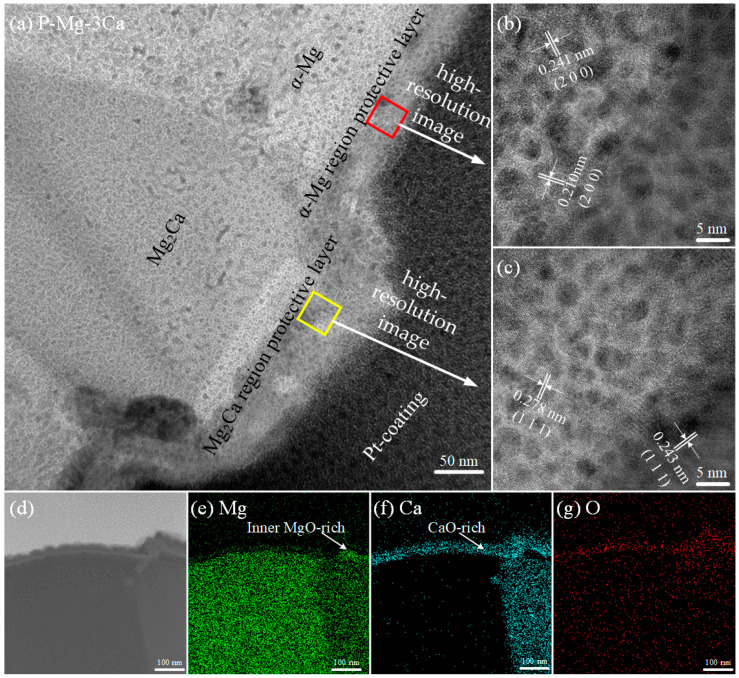
BF TEM images of cross section and element mappings of P-Mg-3Ca preheating at 400 °C for 8 h. (**a**) Cross section, (**b**) high-resolution image of red-boxed area in (**a**), (**c**) high-resolution image of the yellow-boxed area in (**a**), (**d**) STEM image with the corresponding EDS mapping of (**e**) Mg, (**f**) Ca and (**g**) O. Note that the tiny particles observed over the cross-section in (**a**–**c**) are composed of Pt induced by contamination during the continuous thinning process in the FIB sample preparation.

The bright-field (BF) TEM images and high-resolution images of the cross-sectional oxide layer formed in the Mg-hcp region and the eutectic phase Mg_2_Ca region on the P-Mg-3Ca surface at 400 °C for 8 h are shown in Figure 5a–c. The surface is covered with a composite film, and it is divided into two sublayers, which is dark in the outer sublayer and white-grey in the inner sublayer. The thicknesses of the composite film are different on the Mg-hcp region (40 ± 4 nm thick) and the eutectic Mg_2_Ca region (47 ± 8 nm thick), but they both consist of MgO and CaO. Oxide grains of MgO with lattice spacing *d* = 0.210 for (2 0 0) and *d* = 0.243 for (1 1 1), as well as CaO with *d* = 0.241 for (2 0 0) and *d* = 0.278 for (1 1 1), are identified in Figure 5b,c. The STEM images of the cross-section of the protective film with the corresponding element mapping of Mg, Ca and O can be seen in Figure 5d–g. In light of it, the intensity of the Ca signal is strong, especially in the vicinity to the surface, while the Mg signal is somewhat strong only in the inner sublayer. This suggests that the composite protective film on the Mg-hcp region is CaO-rich, while that on the eutectic phase Mg_2_Ca region is MgO-rich in the inner sublayer but CaO-rich in the outer sublayer.

### 3.3. XPS Analysis of P-Mg-3Ca Alloy

**Figure 6:**
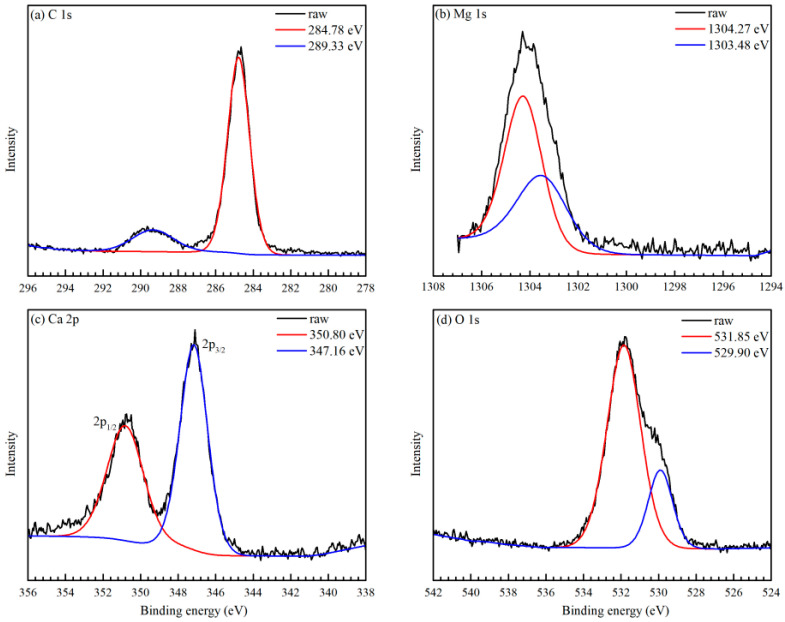
XPS spectra of Mg-3Ca with preheating at 400 °C for 8 h. (**a**) C 1s, (**b**) Mg 1s, (**c**) Ca 2p and (**d**) O 1s.

To characterize the composition of the surface on Mg-3Ca after the preheating, the XPS measurements were carried out, with the results shown in Figure 6. In Figure 6a, the C 1s spectra gives two binding energies at 284.8 eV and 289.33 eV. It is common practice to calibrate the binding energies using a C 1s peak at 284.8 eV as a reference, while the other peak at around 289.33 eV can be assigned as CO_3_^2−^ [32], attributed to CO_2_ contamination in the air. In Figure 6b, the Mg 1s spectra were split into two peaks at 1303.48 eV and 1304.27 eV, which are emitted by metallic Mg and surface oxide layer of MgO, respectively [33]. Figure 6c exhibits two obvious peaks in the spectrum of the Ca 2p orbital at 350.80 eV and 347.16 eV, which are emitted by the ionization of the two fine structure components of Ca 2p1/2 and Ca 2p3/2 [34]. Correspondingly, in Figure 6d, the O1s peak consists of three components. Three different binding energies at 532.30 eV, 531.85 eV and 529.90 eV are attributed to MgO, CO_3_^2−^ and CaO, respectively [7,35]. According to the XPS results, the oxide layer on the surface is deduced to be mainly composed of MgO and CaO.

**Figure 7:**
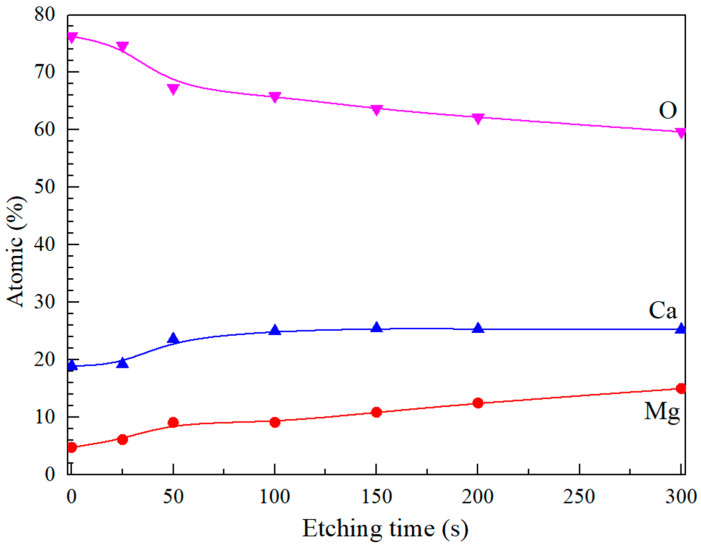
The XPS depth profiles of Mg, Ca and O atomic content along the depth direction in Mg-3Ca alloys preheated at 400 °C for 8 h.

Figure 7 displays the depth profiles of Mg, Ca, O and C elements distributed along the depth direction within the oxide film of P-Mg-3Ca alloy characterized by XPS. It is clear that the simultaneous presence of Mg, Ca and O in the oxide layer indicates the mixed MgO and CaO oxides. The accumulation of Ca due to outward segregation with only a minor content of Mg can be observed at the surface region. The O content is also high at the surface, possibly due to adsorbed oxygen on the sample surface. As the etching process proceeds, the atomic percentages of Mg and Ca increase slightly while the atomic percentage of O decreases. Notably, the ratio of Mg to Ca increases from approximately 1:4 at the surface to 1:2 inside the oxide layer, which is consistent with the TEM results, suggesting that the oxide is mostly Ca-rich near the surface, and Mg is mostly found towards the oxide/alloy interface.

### 3.4. Oxidation Kinetics of uP-Mg-xCa Alloy and P-Mg-xCa Alloy

**Figure 8:**
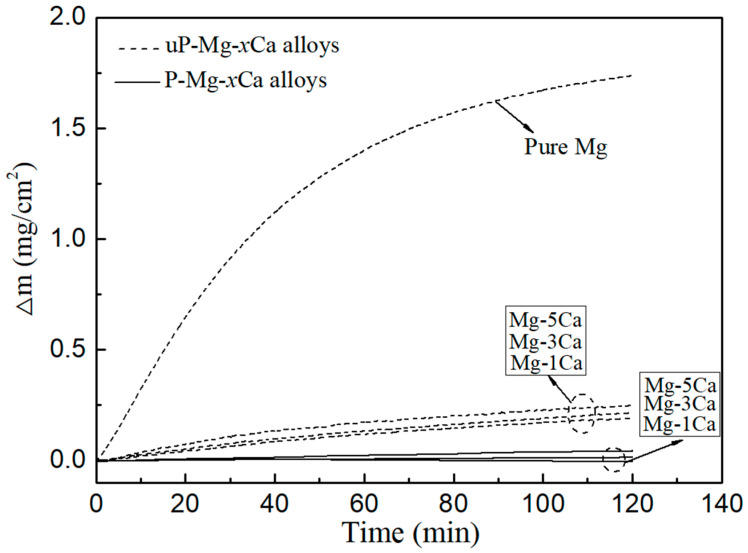
Mass gain curves of oxidation at 400 °C for 2 h for pure Mg, uP-Mg-*x*Ca alloys in dashed and P-Mg-*x*Ca alloys in Ar atmosphere at 400 °C for 8 h in solid.

Figure 8 shows the mass gain during 2 h oxidation in O_2_ at 400 °C for pure Mg and preheated Mg-*x*Ca alloys. The case without preheating is also given for comparison. The mass gain of pure Mg rises rapidly with increasing oxidation time, reaching 1.75 mg/cm^2^ after 2 h. Compared to pure Mg, the mass gains of Mg-*x*Ca samples without the preheating in Ar atmosphere are significantly low, e.g., 0.19 mg/cm^2^, 0.22 mg/cm^2^ and 0.25 mg/cm^2^ for Mg-1Ca, Mg-3Ca and Mg-5Ca, respectively. This indicates that the addition of Ca effectively inhibits the oxidation of Mg alloys. Meanwhile, the mass gains of preheated Mg-*x*Ca at 400 °C are even further reduced, which is only 0.02 g/cm^2^ for Mg-3Ca and 0.05 mg/cm^2^ for Mg-5Ca. This indicates that the oxidation resistance of the preheated Mg-*x*Ca alloy has been further improved. A slight mass loss of the preheated Mg-1Ca is observed during the TGA analysis, which is attributed to the evaporation of Mg. To rule out moisture errors, by fixing the heating rate at 50 °C/min and the O_2_ flow rate at 60 mL/min, all moisture should be evaporated before reaching 400 °C. 

The oxidation kinetic curves of metals can be described by parabolic law, power function law or linear law as shown in Equations (1)–(3) [13,20,29,30]. In order to visualize the difference in oxidation resistance between uP-Mg-*x*Ca and P-Mg-*x*Ca alloys, we fitted the curves in Figure 8, and the results are shown in Table 1. Among them, since the mass gain of P-Mg-1Ca is negative, its results are shown in Table 1.

Δ*m* = (*K_P_t*)^1/2^ (1)

Δ*m* = *At^n^* + *B* (2)

Δ*m* = *K_L_t* + *C* (3)

where Δ*m* represents the mass gain per unit area (mg/cm^2^), *t* represents the oxidation time (min), *K_P_* and *K_L_* represent the parabolic law oxidation rate (mg^2^/cm^4^/min), *A*, *B* and *C* are fitting constants, and *n* is the exponent. The parameters of the oxidation kinetics fitting curves of pure Mg and Mg-*x*Ca alloys are shown in Table 1. *R*^2^ represents the confidence of the fitting curve. When *R*^2^ > 0.8, it can be understood that the fitting curve is credible. 

All curves satisfy the parabolic law with *R*^2^ > 0.8. The *K_P_* of pure Mg is the largest at 0.029 mg^2^/cm^4^/min. In comparison, the *K_P_* of uP-Mg-*x*Ca alloy are significantly smaller by two orders of magnitude. It is noteworthy that the *K_P_* of P-Mg-5Ca and P-Mg-3Ca alloys are reduced by three and four orders of magnitude, respectively. The mass gain curves in Figure 8 correspond to the *K_P_* values in Table 1.

### 3.5. Oxidation Behavior of uP-Mg-xCa Alloy and P-Mg-xCa Alloy

**Figure 9:**
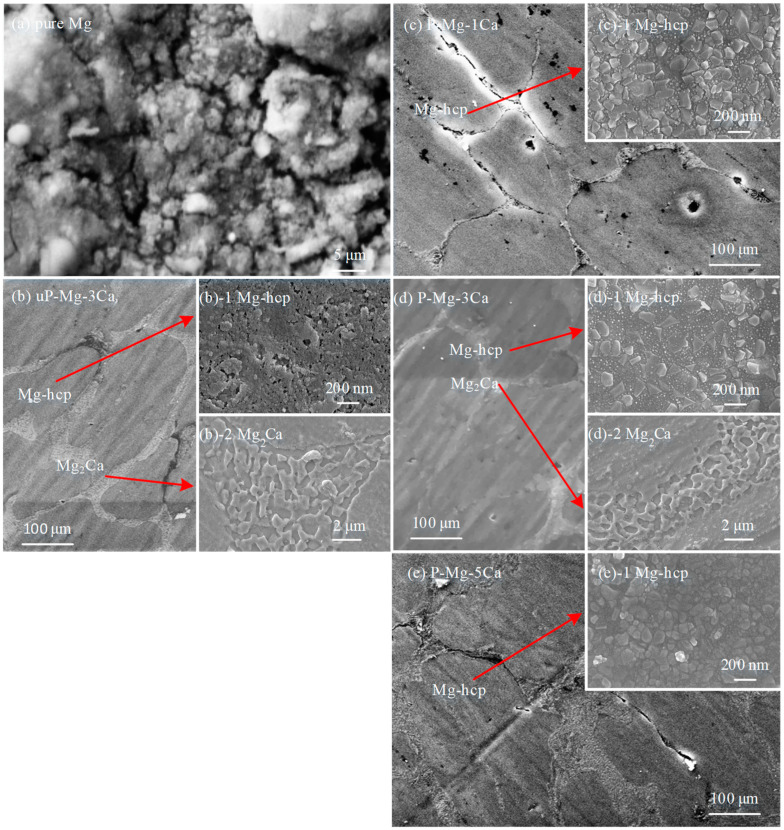
Surface morphology images of pure Mg and Mg-*x*Ca oxidized at 400 °C for 2 h, with SEM images of pure Mg after oxidation in (**a**), uP-Mg-3Ca in (**b**), P-Mg-1Ca in (**c**), P-Mg-3Ca in (**d**) and P-Mg-5Ca in (**e**). The inserts in (**b**–**e**), respectively, exhibit high magnification FESEM images of Mg-hcp of uP-Mg-3Ca in (**b**)-1, P-Mg-1Ca in (**c**)-1, P-Mg-3Ca in (**d**)-1 and P-Mg-5Ca in (**e**)-1, and also those of Mg_2_Ca of uP-Mg-3Ca in (**b**)-2 and P-Mg-3Ca in (**d**)-2.

To characterize the oxide scales on Mg-*x*Ca alloys after oxidation, Figure 9 shows the SEM or FESEM surface morphology of pure Mg, uP-Mg-3Ca, P-Mg-1Ca, P-Mg-3Ca and P-Mg-5Ca after oxidation at 400 °C for 2 h. The surface of pure Mg in Figure 9a is rough with a large number of cracks or pores in it, which should be related to the low PBR of MgO [28]. In comparison, the SEM surface image of oxidized uP-Mg-3Ca in Figure 9b looks much denser and smoother, but the FESEM image in the insert of Figure 9(b)-1 shows that some tiny cracks and pores are still visible on the surface of the main phase of Mg-hcp, indicating that the addition of Ca cannot fully suppress the negative effect of the low PBR of MgO. The reason for this is likely that outward diffusion of Ca is not sufficient to form a protective Ca-rich oxide during oxidation. In contrast, all the surfaces of oxidized P-Mg-1Ca in Figure 9c, P-Mg-3Ca in Figure 9d and P-Mg-5Ca in Figure 9e become dense, which should be attributed to the protective role of the surface MgO-CaO composite film. However, some tiny pores still exist in P-Mg-1Ca but not for the other two, which may have originated from those produced during the preheating process as mentioned above. Furthermore, the FESEM images of the main phase of Mg-hcp in the inserts of Figure 9(c)-1 for P-Mg-1Ca, Figure 9(d)-1 for P-Mg-3Ca and Figure 9(e)-1 for P-Mg-5Ca show that the surfaces are dense, consisting of oxide grains. In comparison, the grain size is largest for P-Mg-3Ca, in agreement with the result that the OR of P-Mg-3Ca is the highest among them in Figure 8. In addition, the FESEM images in the inserts of Figure 9(b)-2 for uP-Mg-3Ca and Figure 9(d)-2 for P-Mg-3Ca are also given, which show that the oxides located at the eutectic structure consist of a discontinuous protruding structure. Note that, in Figure 9c, almost no eutectic structures are observed in the inter-grain spacing between those Mg-hcp grains for P-Mg-1Ca. Instead, cracks apparently formed there, likely due to the low Ca content in P-Mg-1Ca. The difference in the surface morphology of pure Mg, uP-Mg-*x*Ca and P-Mg-*x*Ca after oxidation in Figure 8 suggests that the protective role of the surface oxide layer is the worst for pure Mg but the best for P-Mg-3Ca, which also corresponds well with the change in their oxidation mass gain curves in Figure 8. 

The fact that the OR of the P-Mg-3Ca is higher than that of the P-Mg-5Ca alloys is likely related to the formation of CaO/MgO mixed oxide in the protective films. During the preheating, when Ca is segregated to the surface of P-Mg-*x*Ca, it will be oxidized into CaO. It is reasonable that the grain size of CaO/MgO mixed oxide should be related to the concentration of Ca added into Mg. As demonstrated in the inserts of (d)-1 in Figure 9d and (e)-1 in Figure 9e, the grain size of CaO/MgO mixed oxide at the place of the α-Mg phase for P-Mg-5Ca is somewhat smaller than that of P-Mg-3Ca. As a result, the grain boundary area of CaO/MgO will be larger for P-Mg-5Ca. Since the grain boundary diffusion is much faster than the bulk diffusion, the grain boundary diffusion of O along CaO/MgO will be faster for P-Mg-5Ca than that for P-Mg-3Ca, slightly lowering the OR of P-Mg-5Ca.

**Figure 10:**
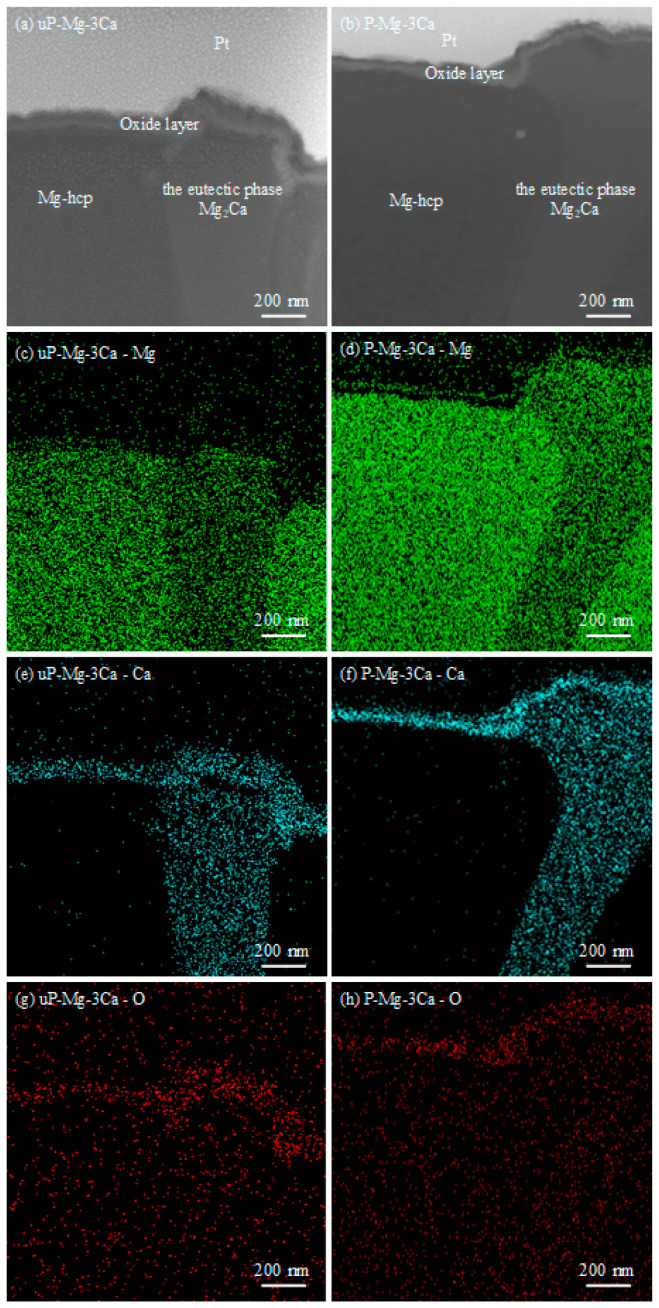
Cross-section STEM morphology of (**a**) uP-Mg-3Ca and (**b**) P-Mg-3Ca oxidized at 400 °C for 2 h with the EDS mapping of Mg in (**c**,**d**), Ca in (**e**,**f**) and O in (**g**,**h**).

To clarify how the eutectic phase Mg_2_Ca would influence the growth of the oxide layer, the cross-sectional TEM images of uP-Mg-3Ca and P-Mg-3Ca oxidized for 2 h at 400 °C are shown in Figure 10 with (a) uP-Mg-3Ca and (b) P-Mg-3Ca, and the corresponding distribution of Mg, O and Ca on the cross-section is shown in Figure 10c–h. In (a,b), the Mg-hcp phase and the eutectic phase Mg_2_Ca can be basically identified with the help of the EDS mapping results in Figure 10c–h, where the Mg signal is weak in the Mg_2_Ca region but strong in the Mg-hcp region. Moreover, a thin oxide layer that formed on uP-Mg-3Ca and P-Mg-3Ca alloys can be clearly observed, where the signals of Ca and O look somewhat strong, but that of Mg seems relatively weak. In Figure 10a, the surface oxide layer on uP-Mg-3Ca at the Mg-hcp and Mg_2_Ca positions are significantly different, which is flat with a thickness of ~105 nm at the Mg-hcp position, while it is about 160 nm thick at the Mg_2_Ca position. After the preheating, the oxide layer thicknesses at the Mg-hcp and Mg_2_Ca position are both uniform as approximately 80 nm for P-Mg-3Ca, as shown in Figure 10b. As measured in Figure 5, the dense and compact MgO/CaO composite protective film formed on P-Mg-*x*Ca during the preheating are 40 ± 4 nm thick in the Mg-hcp region and 47 ± 8 nm thick in the eutectic Mg_2_Ca region. By calculation, it is thus clear that the increases in the thickness during oxidation of Mg-hcp and the eutectic phase Mg_2_Ca are only approximately 40 nm and 33 nm, respectively. This indicates that the preheating process significantly enhances the OR, while the OR at the Mg_2_Ca position after preheating is slightly stronger than that at the Mg-hcp position, consistent with the mass gain measurement in Figure 8 and the surface morphology observation in Figure 9. However, since the OR at the Mg_2_Ca position is weaker without the preheating process (as shown in Figure 10a), it indicates that the preheating process enhances the OR of Mg_2_Ca positions to a more obvious extent.

### 3.6. Mechanism of Protective Film Formation

The chemical reactions that may occur during the preheating of Mg-Ca alloys are described as follows [6,11,19,20]:

2 Mg(s) + O_2_(g) = 2 MgO(s) (4)

2 Ca(s) + O_2_(g) = 2 CaO(s) (5)

Ca(s) + MgO(s) = CaO(s) + Mg(s)(6)

The standard Gibbs free energy of Equations (4) and (5) is calculated using the thermodynamic data from Ref. [36]:

Δ*G*_1_^0^(400 °C) = −1203670 + 218.69T = −1056505.09 (J/mol) (7)

Δ*G*_2_^0^(400 °C) = −1268450 + 206.71T = −1128334.17 (J/mol) (8)

According to the values of Δ*G*_1_^0^ and Δ*G*_2_^0^, the standard Gibbs free energy of Equation (6) is:

Δ*G*_3_^0^(400 °C) = −32390 − 5.99T = −36421.27 (J/mol) (9)

The Gibbs free energy change Δ*G*_3_ for Equation (6) can be calculated by the Equation (10):

(10) $$\Delta G_{3}=\Delta G_{3}^{0}+RTLn\frac{a_{\mathrm{Mg}}\cdot a_{\mathrm{CaO}}}{a_{\mathrm{Ca}}\cdot a_{\mathrm{MgO}}}$$

where *R* is the gas constant, *T* represents the reaction temperature in *K* and *a* represents the activity. Since MgO and CaO are practically pure solids, and they can only have solubility each at 1600 °C, the activities are set to unity with *a*_CaO =_ *a*_MgO_ = 1 [37]. *a*_Ca_ and *a*_Mg_ can be replaced by the corresponding mole fractions [19]. 

The positive and negative values of Δ*G*_3_ can be used to determine the sequence of CaO and MgO formation. Assuming the critical concentration of Ca is *a*_0_, and then if *a*_Ca_ > *a*_0_ (Δ*G*_3_ < 0), CaO could be formed prior to MgO. *a*_0_ is calculated as 3.13 × 10^−4^ at% (0.003 wt.%) with Equation (10). This indicates that CaO is first formed on the surface of the Mg-*x*Ca alloys during the preheating.

**Figure 11:**
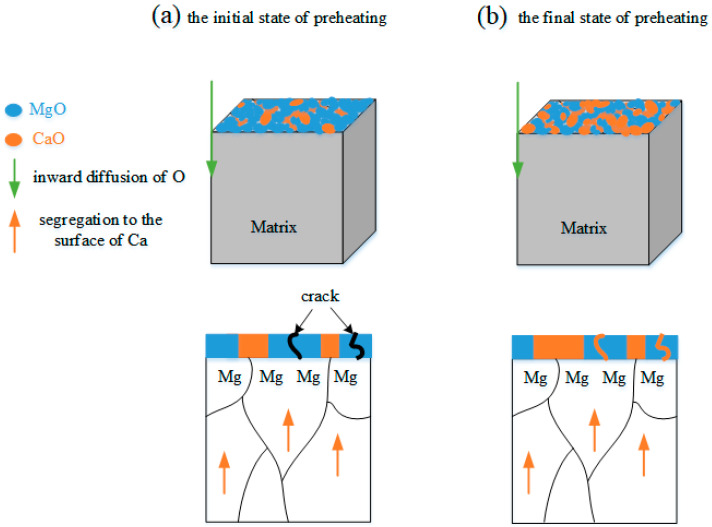
Schematic illustration of the formation of the MgO and CaO composite layer during preheating in Ar atmosphere with 0.1 Pa O_2_ at 400 °C for 8 h.

The mechanism of the protective layer formation is discussed here with the schematic illustration shown in Figure 11. The remnant O_2_ partial pressure in Ar atmosphere during the preheating is about 0.1 Pa at 400 °C, which is higher than decomposition pressure of MgO and CaO, generating MgO and CaO simultaneously, as shown in the initial stage of preheating in Figure 11a. However, the PBR of MgO is 0.81, which indicates that some oxygen still diffuses to the substrate surface through the cracks of MgO. After the preheating process, Ca on the surface is gradually consumed by the oxygen reaction, causing the appearance of Ca-poor areas on the surface. Since Ca is the surface-active element, the Ca-poor regions will drive the Ca dissolved in the matrix to segregate to the surface. Meanwhile, Ca reacts with oxygen to form CaO, filling the cracks of MgO. As a result, a dense and compact composite oxide film generated by CaO and MgO covers the Mg-*x*Ca base, as shown in the final state of preheating in Figure 11b. The composite oxide film formed on the eutectic Mg_2_Ca region is slightly thicker than that on the α-Mg region, which should be ascribed to the large ratio of Ca in Mg_2_Ca relative to α-Mg. As the oxidation process progresses, the dense protective film inhibits the inward diffusion of oxygen and the outward evaporation of Mg, raising the OR of Mg and its alloys [38]. 

## 4. Conclusions

This work investigated the OR of Mg-*x*Ca (*x* = 1, 3 and 5 wt.%) by considering the effect of the preheating treatment. The results indicate that the OR of P-Mg-*x*Ca alloys increases significantly after preheating at 400 °C in Ar atmosphere. The P-Mg-3Ca alloy has a flat surface and no cracks at the boundary of Mg_2_Ca, exhibiting the strongest OR. This is attributed to a dense and compact MgO/CaO composite protective film formed on P-Mg-*x*Ca during preheating, which is 40 ± 4 nm thick in the Mg-hcp region and 47 ± 8 nm thick in the eutectic Mg_2_Ca region. After the preheating process, the OR of the P-Mg-*x*Ca alloy is raised especially for P-Mg-3Ca compared to the case without preheating. Interestingly, the OR at the Mg_2_Ca position after preheating is slightly stronger than that at the Mg-hcp position. As for the observation that the improvement in the OR of Mg-5Ca is less obvious, it should be induced by the diffusion of O atoms through the CaO/MgO boundary in the protective film. 

## Figures and Tables

**Table 1:** Oxidation kinetics parameters 400 °C for 2 h fitted to the parabolic for pure Mg, uP-Mg-*x*Ca alloys and P-Mg-*x*Ca alloys in Ar atmosphere.

Alloys	Oxidation Kinetics	Parameters	*R* ^2^
Pure Mg	parabolic	*K_P_* = 0.029	0.96
uP-Mg-1Ca	parabolic	*K_P_* = 2.52 × 10^−4^	0.92
uP-Mg-3Ca	parabolic	*K_P_* = 3.19 × 10^−4^	0.93
uP-Mg-5Ca	parabolic	*K_P_* = 4.84 × 10^−4^	0.96
P-Mg-3Ca	parabolic	*K_P_* = 1.33 × 10^−6^	0.87
P-Mg-5Ca	parabolic	*K_P_* = 1.15 × 10^−5^	0.86

## Data Availability

The original contributions presented in the study are included in the article, further inquiries can be directed to the corresponding authors.
